# Global health is in crisis: How can Canada contribute?

**DOI:** 10.1371/journal.pgph.0004762

**Published:** 2025-06-11

**Authors:** Prativa Baral, Leah Shipton, Gatien de Broucker, Daniel Eisenkraft Klein

**Affiliations:** 1 Department of Global and Public Health, McGill University School of Population and Global Health, Montréal, Québec, Canada; 2 Department of Health Policy & Management, Johns Hopkins Bloomberg School of Public Health, Baltimore, Maryland, United States of America; 3 Department of Political Science, University of British Columbia, Vancouver, British Columbia, Canada; 4 School of Public Policy, Simon Fraser University, Vancouver, British Columbia, Canada; 5 Department of International Health, Johns Hopkins Bloomberg School of Public Health, Baltimore, Maryland, United States of America; 6 Program On Regulation, Therapeutics, And Law (PORTAL), Department of Medicine, Harvard University, Brigham and Women’s Hospital, Boston, Massachusetts, United States of America; PLOS: Public Library of Science, UNITED STATES OF AMERICA

As the United States pulls back from global health leadership and the United Kingdom cuts back foreign aid [1,2], a large portion of which goes to the health sector, Canada stands at a crossroads. Do we step up, or do we turn inward and abandon global health commitments? We argue the latter is a mistake, based on the false idea that global health happens ‘over there’, and is not connected to domestic health. And like others, we also know that Canada’s approach to global health must change dramatically [3,4].

Over the past two years, we served as the Secretariat for an expert panel on Canada’s role in global health, convened by the Royal Society of Canada and the Canadian Academy of Health Sciences. The panel’s report [5] published in March 2025, is a call to action, but also a reality check. Conversations with stakeholders revealed a sobering disconnect: our global health engagement often lacks coherence, substance, long-term vision, and effective coordination [5]. We were particularly inspired by conversations with fellow early career scholars and practitioners, who are uncompromising in their commitment to imagine and practice an approach to global health that rectifies these shortcomings.

Meanwhile, critical global health conversations are evolving and being led by diverse players including BRICS nations [6], African countries, including Senegal and Rwanda, making strides toward regional vaccine manufacturing [7], and by small states like Barbados shaping the climate-health agenda [8].

Canada is missing in action. But it does not have to be this way.

## No more business as usual

One of the strongest messages we heard during our work is that to make global health relevant again, it is important to stop separating the “global” from the “local.” The view that global health as a distant crisis, an outbreak confined to another country, or a problem requiring solutions from others has undermined Canada’s capacity to lead decisively. In this globalized world, the distinction between global and domestic health is both artificial and counterproductive.

For one, Canada’s pressing public health challenges - the opioid crisis [9], persistent gaps in pandemic preparedness [10], long-standing boil water advisories on First Nations reserves [11], health mis/disinformation campaigns [12], and widespread burnout of health workers [13,14] - are part of a global trend. In other areas, we lag. For example, unlike other countries with universal healthcare systems, Canada has only recently made meaningful strides in expanding a national pharmacare system [15]. How we manage these crises (or ignore them) impacts our credibility as leaders in global public health. Seeing these challenges as global also opens opportunities for collaboration and learning - something many consulted for the report’s development are eager to do.

Global health is also not just about overseas development assistance; it is intimately tied to Canada’s foreign and domestic policy choices, including on trade. For instance, Canada’s judicial, legislative, financial, and diplomatic support for its mining industry overseas persists despite a track record of human rights abuses and longstanding advocacy to regulate the industry to protect against these abuses [16,17]. The international recruitment of health workers to solve our domestic shortages puts health systems and patients in countries with even fewer resources at greater risk [18]. And our continued investment in fossil fuel production contradicts stated commitments to address the climate crisis [19].

## What change is needed?

If Canada wishes to position itself as a credible actor in global health, we need to start by walking the talk. That means following through on our domestic and global commitments to improve health. Domestically, this includes, for example, implementing the recommendations of the Truth and Reconciliation Commission [20], which have direct and indirect impacts on Indigenous peoples’ health. After 13 years, none of the seven health-related calls to action have been fully implemented [21]. This includes Call to Action #18, which urges governments to recognize and address the long-term damaging impacts of previous policies, including residential schools, on Indigenous peoples’ health. Likewise, Call to Action #23 to increase the number of Indigenous health professionals and provide cultural competency training for all healthcare professionals, has yet to be fully realized.

At the same time, strengthening Canada’s credibility in global health means investing in the next generation. This requires the creation of real opportunities for early career professionals to engage in international work that values mutual learning, rather than outdated models of one-way “assistance” [5,22]. This includes training the next generation of leaders through accessible, interdisciplinary education grounded in ethics, governance, and cultural humility.

This commitment must include confronting and reconciling contradictions in our current policies and practices - such as investing in multilateral health initiatives like GAVI while continuing to support industries, including mining and fossil fuels, that directly undermine the health of those very same communities both at home and globally. Crucially, it means funding for global health that isn’t driven by short-term political cycles, branding exercises, or a desire for “reach” over real impact. Our engagement activities for the report repeatedly emphasized that Canada’s contributions to global health must be rooted in long-term partnerships, consistent funding beyond electoral cycles, and genuine engagement with local knowledge. Compensation for lived expertise, particularly for those historically excluded from policy processes, must be an integral part of our approach.

Canada has an opportunity to contribute meaningfully, not because others are failing, but because the complexity and urgency of today’s challenges demand it. We live in an age of turbulence: climate change, pandemics, geopolitical instability, forced displacement, and the ethical risks of emerging technologies. These issues are compounding and deeply interconnected. While these impacts will be most acutely felt by the world’s most vulnerable, Canada is not immune. Our domestic public health systems and our global health architecture are not currently designed for the nimbleness, reactivity, and adaptability required to address these complexities.

Global health is not just a tokenistic gesture of solidarity. It is the infrastructure for our survival. As we head into an uncertain future, the question is not whether Canada will be affected by global health challenges, but whether we will shape the response, or be left reacting to it, five steps too late.

Meaningful leadership requires more than signing global declarations or showing up at global forums. It means taking responsibility, at home and abroad, for the health of people and the planet. Now is the time for Canada to show that global health is not foreign aid nor charity: it is a domestic and global imperative. And it starts right here, at home.
